# Correction: Parity and post-reproductive mortality among U.S. Black and White women: Evidence from the Health and Retirement Study

**DOI:** 10.1371/journal.pone.0319187

**Published:** 2025-02-06

**Authors:** The PLOS One Staff

## Notice of republication

This article was republished on January 29, 2025, to correct error in Fig 1 caption and some minor typographical errors that were introduced during the typesetting process. The publisher apologizes for these errors. Please download the article again to view the correct version. The originally published, uncorrected article and the republished, corrected article are provided here for reference.

## Supporting information

S1 File Originally published, uncorrected article. (PDF)

S2 File Republished, corrected article. (PDF)
