# Supplementary figures and images for: Parity and post-reproductive mortality among U.S. Black and White women: Evidence from the health and retirement study
Source: PLoS One. 2024 Sep 19;19(9):e0310629. doi: 10.1371/journal.pone.0310629 (PMC11412515; doi:10.1371/journal.pone.0310629)

**Table S1. Sample Construction and Data Flow**

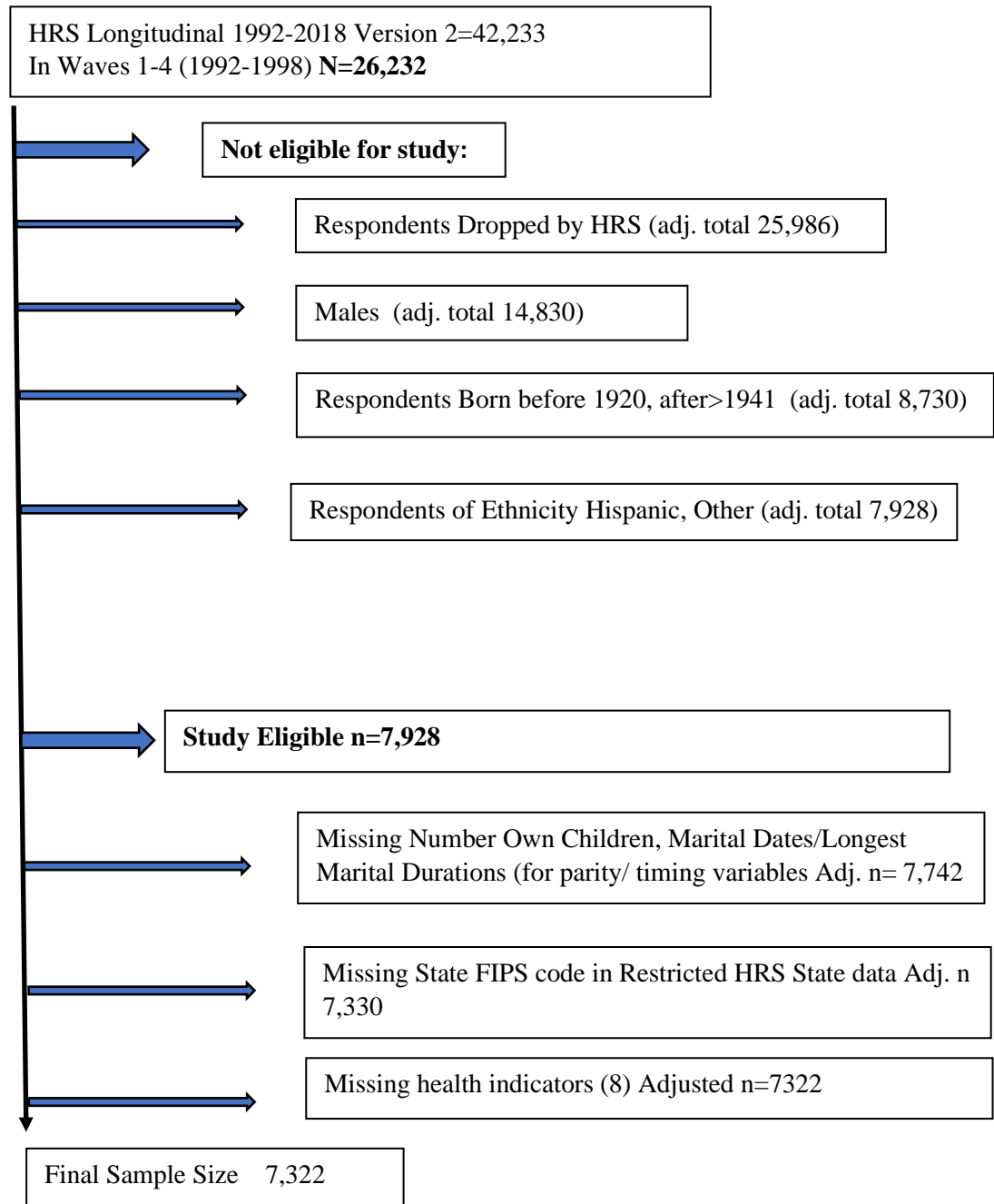

Supplement: S1 Table — (PDF) [file pone.0310629.s001.pdf]
