# Supplementary material for: Parity and post-reproductive mortality among U.S. Black and White women: Evidence from the health and retirement study
Source: PLoS One. 2024 Sep 19;19(9):e0310629. doi: 10.1371/journal.pone.0310629 (PMC11412515; doi:10.1371/journal.pone.0310629)
Supplement: S2 Table — (PDF) [file pone.0310629.s002.pdf]

Table S2. Age-Adjusted Proportional Hazards Models: All-Cause Mortality, Black and White Women

	All Women				*Parous Women*		
	**Model 1**	**Model 2**	**Model 3**	**Model 4**	**Model 5**	**Model 6**	**Model 7**
	HR [95% CI]	HR [95% CI]	HR [95% CI]	HR [95% CI]	HR [95% CI]	HR [95% CI]	HR [95% CI]
Age	1.08***	1.07***	1.08***	1.12***	1.07***	1.08***	1.11***
	[1.06-1.08]	[1.06-1.08]	[1.07-1.10]	[1.11-1.13]	[1.06-1.08]	[1.07-1.09]	[1.09-1.12]
Black Women			1.59***	1.39***		1.47***	1.26*
			[1.33-1.91]	[1.15-1.67]		[1.23-1.76]	[1.05-1.52]
Black Women*Age			0.99*	0.99			
			[0.98-0.99]	[0.98-1.00]			
Reproductive Timing							
Early First Birth					1.18***	1.15***	1.07
					[1.10-1.26]	[1.06-1.24]	[0.99-1.15]
Late First Birth					1.15	1.13	1.01
					[0.83-1.61]	[0.80-1.59]	[0.72-1.43]
Premarital Birth					1.07^{+}	1.11**	1.09*
					[0.99-1.15]	[1.03-1.20]	[1.01-1.17]
Children Ever Born							
Infecundity Probability		1.06***	1.05**	1.03^{+}			
		[1.03-1.10]	[1.02-1.09]	[1.00-1.06]			
Observed 0 Births	1.18**	1.12+	1.15*	1.08			
	[1.06-1.32]	[1.00-1.25]	[1.02-1.30]	[0.95-1.22]			
Observed 1 Birth	1.20**	1.17**	1.12+	1.05	1.18**	1.12+	1.05
	[1.07-1.34]	[1.05-1.32]	[0.99-1.28]	[0.93-1.19]	[1.06-1.33]	[0.99-1.27]	[0.93-1.19]
Observed 3 Births	1.00	1.00	1.01	0.98	0.98	0.99	0.98
	[0.92-1.10]	[0.92-1.10]	[0.92-1.11]	[0.89-1.08]	[0.90-1.08]	[0.90-1.09]	[0.89-1.07]
Observed 4 Births	1.08	1.08	1.08	1.00	1.04	1.04	0.99
	[0.98-1.19]	[0.98-1.19]	[0.97-1.20]	[0.90-1.11]	[0.94-1.14]	[0.94-1.15]	[0.89-1.10]
Observed 5 Births	1.17*	1.17**	1.16*	1.02	1.12^{+}	1.12^{+}	1.00
	[1.04-1.32]	[1.04-1.32]	[1.03-1.32]	[0.90-1.15]	[1.00-1.26]	[0.99-1.26]	[0.89-1.14]
Observed 6+ Births	1.24***	1.24***	1.17**	1.07	1.17**	1.10^{+}	1.05
Reference = 2 Births	[1.12-1.38]	[1.12-1.38]	[1.04-1.31]	[0.95-1.20]	[1.05-1.30]	[0.98-1.24]	[0.93-1.18]
Early Life Course							
Infant Mortality Rate			1.00	1.00		1.00	1.00
			[0.99-1.00]	[0.99-1.00]		[1.00-1.01]	[1.00-1.01]
Born in the South			1.08*	1.00		1.09*	1.03
			[1.01-1.17]	[0.91-1.10]		[1.01-1.18]	[0.93-1.14]
Child Health Poor-Fair			1.27***	1.14*		1.21**	1.10
			[1.13-1.44]	[1.01-1.29]		[1.07-1.38]	[0.97-1.26]
Parent 8th Grade or More			0.86***	0.96		0.86***	0.94
			[0.80-0.93]	[0.89-1.04]		[0.79-.92]	[0.87-1.02]

Table S2. (continued)

	All Women				*Parous Women*		
	**Model 1**	**Model 2**	**Model 3**	**Model 4**	**Model 5**	**Model 6**	**Model 7**
	HR [95% CI]	HR [95% CI]	HR [95% CI]	HR [95% CI]	HR [95% CI]	HR [95% CI]	HR [95% CI]
SES at HRS Baseline							
Less than High School				1.26***			1.25***
				[1.16-1.36]			[1.15-1.36]
Greater than High School				0.85***			0.83***
Reference = High School				[0.77-0.94]			[0.75-0.91]
Resident in South				1.09*			1.05
				[1.01-1.18]			[0.97-1.14]
HH Income (logged)				0.96***			0.96***
				[0.94-0.98]			[0.94-0.98]
Owns House				0.83***			0.83***
				[0.76-0.90]			[0.75-0.91]
Married				0.93*			0.89**
				[0.86-0.99]			[0.82-0.96]
Health Behaviors, Health Status							
Ever Smoked				1.54***			1.58***
				[1.45-1.65]			[1.48-1.70]
Heavy Drinking				1.39***			1.34**
				[1.18-1.63]			[1.12-1.60]
Baseline # Conditions				1.31***			1.31***
				[1.25-1.36]			[1.26-1.37]
Age*Time	1.00***	1.00***	1.00*	NS	1.00***	1.00**	1.00*
Race*Time	-	-	.99***	.99***	-	.99***	.99***
Wald (Sandwich)/df	1758.2/8	1768.6/9	1879.5/18	2470.8/26	1632.5/10	1704.5/18	2361.8/27
N	7322	7322	7322	7322	6667	6667	6667

Note: Models use cluster robust sandwich standard errors and flags for missing child self-rated health and parent education.

+ $p < .10$ $^{*}p < .05$ $^{**}p < .01$ $^{***}p < .001$
