# Supplementary material for: Parity and post-reproductive mortality among U.S. Black and White women: Evidence from the health and retirement study
Source: PLoS One. 2024 Sep 19;19(9):e0310629. doi: 10.1371/journal.pone.0310629 (PMC11412515; doi:10.1371/journal.pone.0310629)
Supplement: S3 Table — (PDF) [file pone.0310629.s003.pdf]

Table S3. Full Age-Stratified Proportional Hazards Models: All-Cause Mortality, Black and White Women

	All Women				*Parous Women*		
	**Model 1**	**Model 2**	**Model 3**	**Model 4**	**Model 5**	**Model 6**	**Model 7**
	HR [95% CI]	HR [95% CI]	HR [95% CI]	HR [95% CI]	HR [95% CI]	HR [95% CI]	HR [95% CI]
Black Women			1.44***	1.25*		1.42***	1.22*
			[1.21-1.71]	[1.05-1.49]		[1.18-1.70]	[1.01-1.45]
Reproductive Timing							
Early First Birth					1.15***	1.14***	1.06
					[1.08-1.24]	[1.05-1.22]	[0.98-1.14]
Late First Birth					1.08	1.09	0.97
					[0.78-1.50]	[0.78-1.52]	[0.69-1.35]
Premarital Birth					1.11**	1.17***	1.15***
					[1.04-1.20]	[1.09-1.27]	[1.07-1.24]
Children Ever Born							
Infecundity Probability		1.08***	1.07***	1.05**			
		[1.04-1.11]	[1.03-1.10]	[1.02-1.08]			
Observed 0 Births	1.19**	1.11+	1.15*	1.09			
	[1.06-1.33]	[0.99-1.25]	[1.01-1.30]	[0.96-1.23]			
Observed 1 Birth	1.21***	1.18**	1.13*	1.08	1.19**	1.14*	1.08
	[1.08-1.36]	[1.05-1.32]	[1.00-1.28]	[0.95-1.22]	[1.06-1.34]	[1.00-1.29]	[0.95-1.22]
Observed 3 Births	1.01	1.01	1.02	1.00	0.99	1.01	0.99
	[0.92-1.11]	[0.93-1.11]	[0.93-1.13]	[0.91-1.10]	[0.91-1.09]	[0.92-1.11]	[0.90-1.09]
Observed 4 Births	1.10*	1.11*	1.10+	1.05	1.06	1.06	1.02
	[1.00-1.22]	[1.00-1.22]	[1.00-1.22]	[0.94-1.16]	[0.96-1.17]	[0.96-1.18]	[0.92-1.14]
Observed 5 Births	1.16*	1.16*	1.16*	1.04	1.12+	1.11	1.01
	[1.03-1.31]	[1.03-1.31]	[1.03-1.32]	[0.91-1.17]	[0.99-1.26]	[0.98-1.26]	[0.89-1.14]
Observed 6+ Births	1.28***	1.28***	1.22***	1.12+	1.20***	1.15*	1.09
Reference = 2 Births	[1.15-1.42]	[1.15-1.42]	[1.08-1.36]	[0.99-1.26]	[1.08-1.34]	[1.02-1.29]	[0.97-1.23]
Early Life Course							
Infant Mortality Rate			1.01***	1.01***		1.01***	1.01***
			[1.00-1.01]	[1.00-1.01]		[1.00-1.01]	[1.01-1.01]
Born in the South			1.02	0.94		1.04	0.98
			[0.95-1.10]	[0.86-1.03]		[0.96-1.12]	[0.88-1.08]
Child Health Poor-Fair			1.28***	1.16*		1.19**	1.08
			[1.13-1.44]	[1.02-1.31]		[1.05-1.36]	[0.95-1.24]
Parent 8th Grade or More			0.84***	0.93+		0.84***	0.92*
			[0.78-0.91]	[0.86-1.00]		[0.78-0.91]	[0.85-0.99]
Adult SES, HRS Baseline							
Less than High School				1.30***			1.27***
				[1.20-1.40]			[1.17-1.38]
Greater than High School				0.82***			0.84***
Reference = High School				[0.75-0.91]			[0.76-0.93]

Table S3. (continued)

	All Women				*Parous Women*		
	**Model 1**	**Model 2**	**Model 3**	**Model 4**	**Model 5**	**Model 6**	**Model 7**
	HR [95% CI]	HR [95% CI]	HR [95% CI]	HR [95% CI]	HR [95% CI]	HR [95% CI]	HR [95% CI]
Resident in South				1.08^{+}			1.04
				[1.00-1.17]			[0.96-1.13]
HH Income (logged)				$.96^{***}$			$.96^{***}$
				[0.94-0.98]			[0.94-0.98]
Owns House				0.82^{***}			0.82^{***}
				[0.75-0.89]			[0.74-0.90]
Married				0.91^{**}			0.90^{**}
				[0.84-0.98]			[0.83-0.97]
Health Factors							
Ever Smoked				1.52^{***}			1.55^{***}
				[1.42-1.62]			[1.45-1.66]
Heavy Drinking				1.44^{***}			1.39^{***}
				[1.24-1.68]			[1.17-1.65]
Baseline # Conditions				1.22^{***}			1.23^{***}
				[1.17-1.27]			[1.18-1.28]
Race*Time	-	-	$.99^{***}$	$.99^{***}$	-	$.99^{***}$	$.99^{***}$
			[1.00-1.00]	[1.00-1.00]		[1.00-1.00]	[1.00-1.00]
Wald (Sandwich)/df	35.1/6	55.6/7	480.4/15	1040.0/24	71.0/8	471.5/16	1007.4/25
N	7322	7322	7322	7322	6667	6667	6667

Note: Models use cluster robust sandwich standard errors and flags for missing child self-rated health and parent education.

$+ p < .10$ $^{*} p < .05$ $^{**} p < .01$ $^{***} p < .001$
