# Supplementary material for: Parity and post-reproductive mortality among U.S. Black and White women: Evidence from the health and retirement study
Source: PLoS One. 2024 Sep 19;19(9):e0310629. doi: 10.1371/journal.pone.0310629 (PMC11412515; doi:10.1371/journal.pone.0310629)
Supplement: S4 Table — (PDF) [file pone.0310629.s004.pdf]

Table S4. Full Age-Stratified Proportional Hazards Models: All-Cause Mortality, Black Women

	All Women				*Parous Women*		
	**Model 1**	**Model 2**	**Model 3**	**Model 4**	**Model 5**	**Model 6**	**Model 7**
	HR [95% CI]	HR [95% CI]	HR [95% CI]	HR [95% CI]	HR [95% CI]	HR [95% CI]	HR [95% CI]
Reproductive Timing							
Early First Birth					1.04	1.13	1.07
					[0.88-1.24]	[0.94-1.35]	[0.89-1.29]
Late First Birth					0.92	0.92	0.77
					[0.46-1.84]	[0.44-1.95]	[0.34-1.74]
Premarital Birth					1.04	1.03	1.00
					[0.89-1.22]	[0.87-1.22]	[0.84-1.17]
Children Ever Born							
Infecundity Probability		1.06*	1.08**	1.06*			
		[1.01-1.11]	[1.02-1.13]	[1.01-1.11]			
Observed 0 Births	1.34*	1.28+	1.14	1.16			
	[1.02-1.78]	[0.97-1.70]	[0.83-1.55]	[0.84-1.58]			
Observed 1 Birth	1.03	1.01	0.95	0.94	1.03	0.97	0.94
	[0.78-1.36]	[0.76-1.33]	[0.70-1.27]	[0.70-1.25]	[0.77-1.36]	[0.72-1.31]	[0.70-1.27]
Observed 3 Births	0.97	0.96	0.90	0.95	0.97	0.91	0.93
	[0.75-1.25]	[0.74-1.25]	[0.69-1.19]	[0.72-1.25]	[0.75-1.25]	[0.68-1.20]	[0.71-1.23]
Observed 4 Births	1.04	1.06	0.97	0.96	1.04	0.95	0.95
	[0.79-1.38]	[0.80-1.40]	[0.73-1.30]	[0.72-1.27]	[0.78-1.37]	[0.71-1.27]	[0.71-1.26]
Observed 5 Births	1.06	1.08	1.07	0.98	1.05	1.02	0.95
	[0.79-1.41]	[0.81-1.44]	[0.80-1.43]	[0.72-1.32]	[0.78-1.40]	[0.76-1.36]	[0.70-1.29]
Observed 6+ Births	1.23+	1.27*	1.20	1.06	1.21	1.12	1.05
Reference = 2 Births	[0.98-1.55]	[1.01-1.60]	[0.94-1.53]	[0.83-1.36]	[0.96-1.53]	[0.87-1.44]	[0.81-1.35]
Early Life Course							
Infant Mortality Rate			1.01*	1.01*		1.01**	1.02***
			[1.00-1.02]	[1.00-1.02]		[1.00-1.02]	[1.01-1.02]
Born South			0.75**	0.71**		0.76*	0.75*
			[0.61-0.93]	[0.56-0.90]		[0.60-0.96]	[0.59-0.97]
Child Health Poor-Fair			1.38*	1.24		1.27	1.15
			[1.06-1.78]	[0.96-1.59]		[0.95-1.70]	[0.87-1.53]
Parent 8th Grade or More			0.87	0.98		0.93	1.04
			[0.74-1.02]	[0.83-1.16]		[0.78-1.11]	[0.88-1.25]
Adult SES, HRS Baseline							
Less than High School				1.37***			1.31**
				[1.15-1.63]			[1.08-1.58]
Greater than High School				0.93			0.98
Reference = High School				[0.71-1.21]			[0.74-1.30]

Supplement Table S4. (continued)

	All Women				*Parous Women*		
	**Model 1**	**Model 2**	**Model 3**	**Model 4**	**Model 5**	**Model 6**	**Model 7**
	HR [95% CI]	HR [95% CI]	HR [95% CI]	HR [95% CI]	HR [95% CI]	HR [95% CI]	HR [95% CI]
Lives in South				1.05			0.95
				[0.89-1.25]			[0.80-1.14]
HH Income (logged)				0.98			0.97
				[0.94-1.01]			[0.93-1.01]
Owns House				0.90			0.93
				[0.76-1.07]			[0.77-1.11]
Married				0.99			0.91
				[0.84-1.17]			[0.76-1.10]
Health Factors							
Ever Smoked				1.28**			1.28**
				[1.10-1.49]			[1.09-1.51]
Heavy Drinking				1.34			1.21
				[0.71-2.54]			[0.56-2.54]
Baseline # Conditions				1.32***			1.35***
				[1.22-1.43]			[1.24-1.46]
Wald (Sandwich)/df	10.1/6 (NS)	15.0/7	102.8/13	218.41/22	6.8/8(NS)	99.2/14	216.2/23
N	1274	1274	1274	1274	1140	1140	1140

Note: Models use cluster robust sandwich standard errors and flags for missing child self-rated health and parent education.

+ $p < .10$ * $p < .05$ ** $p < .01$ *** $p < .001$
