# Supplementary material for: Parity and post-reproductive mortality among U.S. Black and White women: Evidence from the health and retirement study
Source: PLoS One. 2024 Sep 19;19(9):e0310629. doi: 10.1371/journal.pone.0310629 (PMC11412515; doi:10.1371/journal.pone.0310629)
Supplement: S5 Table — (PDF) [file pone.0310629.s005.pdf]

Table S5. Full Age-Stratified Proportional Hazards Models: All-Cause Mortality, White Women

	All Women				*Parous Women*		
	**Model 1**	**Model 2**	**Model 3**	**Model 4**	**Model 5**	**Model 6**	**Model 7**
	HR [95% CI]	HR [95% CI]	HR [95% CI]	HR [95% CI]	HR [95% CI]	HR [95% CI]	HR [95% CI]
Reproductive Timing							
Early First Birth					1.17**	1.13**	1.05
					[1.08-1.26]	[1.04-1.23]	[0.97-1.14]
Late First Birth					1.12	1.15	1.05
					[0.80-1.57]	[0.80-1.64]	[0.73-1.49]
Premarital					1.11**	1.21***	1.19***
					[1.03-1.20]	[1.12-1.32]	[1.10-1.30]
Children Ever Born							
Infecundity Probability		1.07**	1.07**	1.06*			
		[1.02-1.12]	[1.02-1.12]	[1.01-1.11]			
Observed 0 Births	1.13*	1.07	1.14+	1.10			
	[1.00-1.28]	[0.94-1.22]	[1.00-1.30]	[0.92-1.20]			
Observed 1 Birth	1.24***	1.22**	1.18*	1.10	1.22**	1.17*	1.10
	[1.09-1.40]	[1.08-1.38]	[1.02-1.35]	[0.96-1.26]	[1.08-1.38]	[1.02-1.34]	[0.96-1.26]
Observed 3 Births	1.01	1.02	1.04	1.00	0.99	1.02	1.00
	[0.92-1.12]	[0.92-1.12]	[0.94-1.15]	[0.91-1.11]	[0.90-1.10]	[0.92-1.12]	[0.90-1.10]
Observed 4 Births	1.10+	1.11+	1.12*	1.05	1.06	1.08	1.03
	[0.99-1.22]	[1.00-1.23]	[1.01-1.25]	[0.94-1.18]	[0.95-1.18]	[0.96-1.20]	[0.92-1.15]
Observed 5 Births	1.16*	1.16*	1.18*	1.03	1.11	1.13+	1.01
	[1.01-1.32]	[1.01-1.33]	[1.03-1.36]	[0.90-1.19]	[0.97-1.27]	[0.98-1.30]	[0.88-1.16]
Observed 6+ Births	1.18*	1.18**	1.19**	1.12	1.12+	1.13+	1.10
Reference = 2 Births	[1.04-1.34]	[1.04-1.34]	[1.04-1.37]	[0.98-1.29]	[0.99-1.28]	[0.99-1.30]	[0.96-1.26]
Early Life Course							
Infant Mortality Rate			1.01***	1.01***		1.01***	1.01***
			[1.00-1.01]	[1.00-1.01]		[1.00-1.01]	[1.00-1.01]
Born in the South			1.07	1.00		1.08	1.00
			[0.99-1.16]	[0.90-1.10]		[0.99-1.17]	[0.90-1.11]
Child Health Poor-Fair			1.24**	1.12		1.17*	1.06
			[1.08-1.42]	[0.98-1.30]		[1.01-1.35]	[0.92-1.24]
Parent 8th Grade or More			0.84***	0.92+		0.82***	0.90*
			[0.77-0.91]	[0.85-1.10]		[0.75-0.90]	[0.82-0.98]
Adult SES, HRS Baseline							
Less than High School				1.29***			1.28***
				[1.18-1.42]			[1.17-1.41]
Greater than High School				0.81***			0.82***
Reference = High School				[0.73-0.89]			[0.73-0.91]

Supplement Table S5. (continued)

	All Women				*Parous Women*		
	**Model 1**	**Model 2**	**Model 3**	**Model 4**	**Model 5**	**Model 6**	**Model 7**
	HR [95% CI]	HR [95% CI]	HR [95% CI]	HR [95% CI]	HR [95% CI]	HR [95% CI]	HR [95% CI]
Lives in South				1.06			1.05
				[0.97-1.16]			[0.96-1.16]
HH Income (logged)				0.96**			0.96**
				[0.93-0.98]			[0.93-0.98]
Owns House				0.78***			0.78***
				[0.70-0.86]			[0.70-0.86]
Married				0.88**			0.90*
				[0.82-0.96]			[0.82-0.98]
Health Behaviors, Health Status							
Ever Smoked				1.57***			1.61***
				[1.46-1.70]			[1.49-1.73]
Heavy Drinking				1.46***			1.40***
				[1.24-1.71]			[1.18-1.67]
Baseline # conditions				1.19***			1.20***
				[1.14-1.25]			[1.14-1.26]
Wald (Sandwich)/df	19.3/6	27.1/7	338.8/13	794.3/22	53.8/8	347.2/14	792.8/23
N	6048	6048	6048	6048	5527	5527	5527

Note: Models use cluster robust sandwich standard errors and flags for missing child self-rated health and parent education.

$+p < .10$ $^{*}p < .05$ $^{**}p < .01$ $^{***}p < .001$
