# Supplementary material for: Parity and post-reproductive mortality among U.S. Black and White women: Evidence from the health and retirement study
Source: PLoS One. 2024 Sep 19;19(9):e0310629. doi: 10.1371/journal.pone.0310629 (PMC11412515; doi:10.1371/journal.pone.0310629)
Supplement: S6 Table — (PDF) [file pone.0310629.s006.pdf]

Table S6. Reproductive Timing Sensitivity Analysis: Stratified PH Models: All-Cause Mortality, Black and White Women

	All Parous Women (Table 3)			*Parous Black Women (Table 4)*			*Parous White Women (Table 5)*		
	**Model 5**	**Model 6**	**Model 7**	**Model 5**	**Model 6**	**Model 7**	**Model 5**	**Model 6**	**Model 7**
	HR [95%CI]	HR [95%CI]	HR [95%CI]	HR [95%CI]	HR [95%CI]	HR [95%CI]	HR [95%CI]	HR [95%CI]	HR [95%CI]
Black Women		1.40^{***} [1.17-1.68]	1.21^{*} [1.01-1.45]	-	-	-	-	-	-
Reproductive Timing									
Early First Birth	1.15^{***} [1.08-1.24]	1.14^{***} [1.06-1.22]	1.06 [0.98-1.14]	1.06 [0.89-1.26]	1.14 [0.95-1.37]	1.08 [0.90-1.30]	1.17^{***} [1.08-1.26]	1.13^{**} [1.04-1.23]	1.05 [0.97-1.14]
Late First Birth	1.08 [0.78-1.49]	1.08 [0.78-1.51]	0.97 [0.69-1.36]	0.90 [0.46-1.78]	0.90 [0.43-1.89]	0.76 [0.33-1.72]	1.12 [0.80-1.57]	1.15 [0.81-1.64]	1.05 [0.73-1.49]
Premarital Birth	1.11^{**} [1.03-1.19]	1.17^{***} [1.09-1.26]	1.15^{***} [1.07-1.24]	1.04 [0.88-1.21]	1.02 [0.87-1.21]	0.99 [0.83-1.17]	1.11^{**} [1.03-1.20]	1.21^{***} [1.12-1.32]	1.19^{***} [1.09-1.30]
Flag: Children born ≠ Family File Children[1]	1.13^{**} [1.04-1.22]	1.11^{*} [1.02-1.20]	1.04 [0.96-1.14]	1.17 [0.99-1.37]	1.17 [0.99-1.38]	1.08 [0.91-1.29]	1.09 [0.99-1.20]	1.08 [0.98-1.19]	1.04 [0.94-1.14]
Children Born									
Observed 1 Birth	1.19^{**} [1.06-1.33]	1.14^{*} [1.01-1.28]	1.08 [0.95-1.22]	1.05 [0.79-1.39]	0.99 [0.74-1.35]	0.96 [0.71-1.29]	1.22^{**} [1.07-1.38]	1.17^{*} [1.02-1.34]	1.10 [0.95-1.26]
Observed 3 Births	0.99 [0.90-1.08]	1.01 [0.91-1.10]	0.99 [0.90-1.09]	0.95 [0.73-1.23]	0.90 [0.67-1.19]	0.92 [0.70-1.22]	0.99 [0.90-1.09]	1.01 [0.92-1.12]	0.99 [0.90-1.10]
Observed 4 Births	1.05 [0.95-1.16]	1.05 [0.95-1.16]	1.02 [0.92-1.13]	1.02 [0.77-1.35]	0.93 [0.70-1.25]	0.94 [0.70-1.25]	1.05 [0.94-1.17]	1.07 [0.96-1.19]	1.03 [0.92-1.15]
Observed 5 Births	1.09 [0.96-1.23]	1.09 [0.97-1.24]	1.00 [0.88-1.14]	1.02 [0.76-1.36]	0.99 [0.74-1.34]	0.95 [0.70-1.29]	1.09 [0.97-1.25]	1.11 [0.97-1.28]	1.00 [0.87-1.15]
Observed 6+ Births Ref. 2 Births	1.16^{**} [1.04-1.30]	1.12+ [1.00-1.26]	1.08 [0.96-1.21]	1.15 [0.90-1.47]	1.07 [0.82-1.39]	1.02 [0.79-1.33]	1.10 [0.96-1.25]	1.11 [0.97-1.27]	1.08 [0.94-1.24]
Parity χ^2 Group	**	NS	NS	NS	NS	NS	*	NS	NS
Wald Sandwich/df	81.9 / 9	482.3 /17	1008.7 /26	11.3 /9	100.4 /15	217.1 /24	57.5 /9	354 /15	791 /24
N	6667	6667	6667	1140	1140	1140	5527	5527	5527

[1] Flag=1 denotes Respondent's reported children ever born is unequal to RAND Family Respondent File own in-contact alive children when surveyed.
Note: All models use cluster robust sandwich standard errors. Models 6 adjust for birthplace, childhood health, family SES; Models 7 further adjust for adult baseline SES, health behaviors, marital status and health status.

$^{*}p$<.05 $^{**}p$<.01 $^{***}p$<. 001 (two-tailed tests)
