# Supplementary material for: Parity and post-reproductive mortality among U.S. Black and White women: Evidence from the health and retirement study
Source: PLoS One. 2024 Sep 19;19(9):e0310629. doi: 10.1371/journal.pone.0310629 (PMC11412515; doi:10.1371/journal.pone.0310629)
Supplement: Attachment — Submitted filename: Response to reviewer 9-3-24 Final with table.pdf [file pone.0310629.s008.pdf]

To the Editor and Reviewer:

We are grateful to have the opportunity to respond to the one remaining Reviewer concern about our study. We describe this issue below and how we addressed the concern raised.

The original concern of the Reviewer was:

> "14. Data. Regarding the observed parity and timing indicators (lines 301-311), **age at first birth is calculated using the age of participant and the age of their first child**. This implies that respondents were asked for the ages of all their children, including the age that any who had died would be had they lived. This might be subject to more error **than the ages of living children, and it would be good to have an assessment of the effect of this on results.**" (our bold)

Our original response was:

> Although the HRS did query respondents about their number of children, it did not ask for the age(s) of all children born or list dates of all children born. Rather, it only asked them to report the age of their oldest and youngest living child with whom they were in contact in the baseline interview....

We appreciate the chance to clarify our initial response to the Reviewer's original concern. We apologize; we should have made it clearer that the age of birth variables that we used were indeed based on <u>living children in-contact with the respondent</u>, as obtained from the RAND Family Respondent File. The HRS did not ask respondents to estimate the age that any who had died would be had they lived.

Please let us further note that there is a measurement gap in HRS/RAND data, regarding: (1) parity/number of children ever born; and (2) number of own in-contact living children as ascertained in late-life (at baseline). Regarding the former, HRS/AHEAD directly queried respondents at intake about their number of children born (RAND variable, RAEVBRN). Regarding the latter, the RAND Family File compiled <u>indirect</u> information about respondents' number of own currently-living children from household rosters (Family File variable, R_OWNKIDKN) that captured children living in households, and respondent responses to queries about children living outside of households. HRS/AHEAD respondents were never asked to report their number of <u>children surviving</u>, in the context of their number of children ever born (RAEVBRN).

We reiterate, in response to the Reviewer's initial concern, that we calculated age at birth variables based on the living (surviving) oldest child age reported in the RAND Family File.

In response to our initial response, the Reviewer raised a further concern:

> "I appreciate you including this information, but I still think it is worth including a brief assessment of the possible effect of this on results. **Clearly if their oldest or youngest**

> **child had died (or they had lost contact with them), then ages at first or last birth will be over or under-stated**. Given that **it is possible that the risk of child death is correlated with maternal health**, how might this affect results?"

We address this new concern here.

The Reviewer is concerned that **age at birth will be over or under-stated if an oldest/youngest child had died (or lost contact)**. This is an interesting and important possibility to consider. However, as noted above, HRS/RAND data do not provide respondents' total number of child deaths or the deaths/death dates of any children, in or out of the household. As the Reviewer notes, among alive in-contact children in the RAND Family File, one does not know the true birth order of the reported child (i.e., whether the oldest/youngest child was truly so). One also does not know whether a gap between women's number of children born (RAEVBRN) and their number of later-life in-contact own living children (R_OWNKIDKN) captures birth child(ren) who: died in infancy or in adulthood; were given up at birth for adoption (not uncommon in these birth cohorts); lost contact due to moving overseas (pre-internet, satellite world) or due to parent-child conflict.

With this recognition, we developed perhaps the only test possible of whether the use of RAND Family Respondent File age of oldest living child might introduce error in findings, contingent on whether even earlier-born child(ren) had died/lived/kept contact. We created a Flag variable, using both the RAEVBRN number of children ever born and RAND Family File variable (R_OWNKIDKN) number of own alive, in-contact children in late-life (at baseline), coded as follows:

Flag=1 A respondent is coded 1 if her reported number of children born *differs* from her number of own in-contact living children as compiled in the RAND Family File.

Flag=0 A respondent is coded 0 if her reported number of children born *is equal to (*matches) the number of own in-contact living children as compiled in the RAND Family File.

Again, HRS/RAND data do not allow us to examine birth/survival data for each child born; deaths/death dates are not reported. But we believe that the Flag at least partly addresses the question of whether age of birth findings might be contingent on whether a possible "true" first/oldest child died/lost contact. Such cases would be included in the Flag=1 subgroup. We caution however that, among women whose number of children born is not equal to their number of in-contact alive children in late-life (Flag=1), missing children) could still be of any birth order (not just oldest or youngest).

Our sensitivity analysis (see attached table) adds this Flag to Models for parous women (Models 5-7) in Tables 3-5. The Flags are not necessary in the nulliparous/parous (or all-women) parity models (Tables 3-5, Models 1-4) because they do not include the age at birth variables: nulliparous women have missing values for all age at birth measures. Our statistical findings in

the attached table show that virtually all age at first birth hazard ratios (Models 5-7, Tables 3-5) do not change in strength or significance. Therefore, the answer is "No" to the question of whether coding of *Age of Birth,* using the live, in-contact children matters for findings; findings do not differ when we account for kids who had died/lost contact (i.e., the gap between RAEVBRN and R_OWNKIDKN). But better (different) data are required to ascertain directly whether **it is possible that the risk of child death is correlated with maternal health** or longevity. HRS respondents' children ever born (RAEVBRN) could be "gone" by later life due to social, interpersonal, or health/survival processes. The data do not allow testing of these pathways.

Changes we made: we added the attached sensitivity analysis to the Appendix, as Supplemental Table S6, and added the following to the Analytical Strategy section, starting on Line 503:

> Information about children ever born, harmonized in RAND, was directly reported by HRS/AHEAD participants. In contrast, the RAND Family Respondent File ascertained participants' number of in-contact, alive children from household and participant records. As HRS/RAND data do not report children surviving in the context of children ever born, and RAND Family data do not report deaths/death dates of children who died prior to HRS/AHEAD at baseline, there is a measurement gap: given the universe of children ever born, we only have information about surviving children in late-life (RAND Family Respondent File). This gap suggests that, among parous women, age of oldest child in RAND Family data may be mis-stated. This could introduce error in age at birth findings, contingent on whether even earlier-born child(ren) had died/lost contact (i.e., were not present in the RAND Family roster). In a sensitivity analysis (see Supplement Table S6), we examined possible measurement error in age at first birth variables used in Models for parous women by creating a Flag variable that differentiated number of children ever born and number of own alive, in-contact children in late-life. The Flag=1 applied if women's number of children born *differed* from their number of in-contact, living children in late-life or =0 if the number of children *matched*. We found that statistical results (the size and significance of hazard ratios) for age at first birth variables did not change when Flags were included in Models.

Again, we are grateful for the careful attention paid to our paper and for the chance to revise it. We believe we have been able to respond to the concerns raised and that our paper is stronger as a result.

Supplement Table S6. Reproductive Timing Sensitivity Analysis: Stratified Proportional Hazards Models of Mortality

	All Parous Women (Table 3)			*Black Parous Women (Table 4)*			*White Parous Women (Table 5)*		
	**Model 5**	**Model 6**	**Model 7**	**Model 5**	**Model 6**	**Model 7**	**Model 5**	**Model 6**	**Model 7**
	HR [95%CI]	HR [95%CI]	HR [95%CI]	HR [95%CI]	HR [95%CI]	HR [95%CI]	HR [95%CI]	HR [95%CI]	HR [95%CI]
Black Women		1.40***	1.21*	-	-	-	-	-	-
		[1.17-1.68]	[1.01-1.45]						
Reproductive Timing									
Early First Birth	1.15***	1.14***	1.06	1.06	1.14	1.08	1.17***	1.13**	1.05
	[1.08-1.24]	[1.06-1.22]	[0.98-1.14]	[0.89-1.26]	[0.95-1.37]	[0.90-1.30]	[1.08-1.26]	[1.04-1.23]	[0.97-1.14]
Late First Birth	1.08	1.08	0.97	0.90	0.90	0.76	1.12	1.15	1.05
	[0.78-1.49]	[0.78-1.51]	[0.69-1.36]	[0.46-1.78]	[0.43-1.89]	[0.33-1.72]	[0.80-1.57]	[0.81-1.64]	[0.73-1.49]
Premarital Birth	1.11**	1.17***	1.15***	1.04	1.02	0.99	1.11**	1.21***	1.19***
	[1.03-1.19]	[1.09-1.26]	[1.07-1.24]	[0.88-1.21]	[0.87-1.21]	[0.83-1.17]	[1.03-1.20]	[1.12-1.32]	[1.09-1.30]
Flag: Children born ≠	1.13**	1.11*	1.04	1.17	1.17	1.08	1.09	1.08	1.04
Family File Children[1]	[1.04-1.22]	[1.02-1.20]	[0.96-1.14]	[0.99-1.37]	[0.99-1.38]	[0.91-1.29]	[0.99-1.20]	[0.98-1.19]	[0.94-1.14]
Children Born									
Observed 1 Birth	1.19**	1.14*	1.08	1.05	0.99	0.96	1.22**	1.17*	1.10
	[1.06-1.33]	[1.05-1.32]	[0.95-1.22]	[0.79-1.39]	[0.74-1.35]	[0.71-1.29]	[1.07-1.38]	[1.02-1.34]	[0.95-1.26]
Observed 3 Births	0.99	1.01	0.99	0.95	0.90	0.92	0.99	1.01	0.99
	[0.90-1.08]	[0.91-1.10]	[0.90-1.09]	[0.73-1.23]	[0.67-1.19]	[0.70-1.22]	[0.90-1.09]	[0.92-1.12]	[0.90-1.10]
Observed 4 Births	1.05	1.05	1.02	1.02	0.93	0.94	1.05	1.07	1.03
	[0.95-1.16]	[0.95-1.16]	[0.92-1.13]	[0.77-1.35]	[0.70-1.25]	[0.70-1.25]	[0.94-1.17]	[0.96-1.19]	[0.92-1.15]
Observed 5 Births	1.09	1.09	1.00	1.02	0.99	0.95	1.09	1.11	1.00
	[0.96-1.23]	[0.96-1.24]	[0.88-1.14]	[0.76-1.36]	[0.74-1.34]	[0.70-1.29]	[0.97-1.25]	[0.97-1.28]	[0.87-1.15]
Observed 6+ Births	1.16**	1.14+	1.08	1.15	1.07	1.02	1.10	1.11	1.08
Ref. 2 Births	[1.04-1.30]	[1.00-1.27]	[0.96-1.21]	[0.90-1.47]	[0.82-1.39]	[0.79-1.33]	[0.96-1.25]	[0.97-1.27]	[0.94-1.24]
Parity χ^2 Group	**	NS	NS	NS	NS	NS	*	NS	NS
Wald Sandwich/df	81.9 / 9	482.3 /17	1008.7 /26	71.0 /8	471.5 /16	1007.4 /25	57.5 /6	354 /15	791 /24
N	6667	6667	6667	1140	1140	1140	5527	5527	5527

[1] Flag=1 denotes Respondent's reported children ever born is unequal to RAND Family Respondent File own in-contact alive children when surveyed.
Note: All models use cluster robust sandwich standard errors. Models 6 adjust for birthplace, childhood health, family SES; Models 7 further adjust for adult baseline SES, health behaviors, marital status and health status.
$^{*}p<.05$ $^{**}p<.01$ $^{***}p<.001$ (two-tailed tests)
