# Supplementary material for: Parity and post-reproductive mortality among U.S. Black and White women: Evidence from the health and retirement study
Source: PLoS One. 2024 Sep 19;19(9):e0310629. doi: 10.1371/journal.pone.0310629 (PMC11412515; doi:10.1371/journal.pone.0310629)
Supplement: Attachment — Submitted filename: Combined Reviewer response Final.docx [file pone.0310629.s007.docx]

To the Editor and Reviewers:

We are grateful for the careful attention that both reviewers paid to our paper and for the chance to revise it. In response to the reviewers’ comments, we streamlined the front-end of the paper and updated the Tables presented in the Results section. Additionally, in response to Reviewer 1’s comments, we changed all references to “race” to “race/ethnicity” or “race/ethnic,” made our hypotheses more explicit, and enhanced our discussion of the health policy and research implications of our study. In response to Reviewer 2’s comments, we better defined and differentiated infecundity and fertility, added discussion of anomalous childbearing and marital behaviors of 1920-1941 birth cohorts associated with the historical baby boom generation, revised the description of count regression methods for the analytic strategy section, and responded to specific variable coding issues.

Below, we provide detailed responses to each of the Reviewers’ comments. We believe that we have responded to all of their comments and that our paper is stronger as a result. We are hopeful that our revised paper will be deemed suitable for publication in *PLOS One*. If additional changes are required prior to acceptance, we would welcome the opportunity to make them.

All references below are included in the bibliography of the paper.

**Reviewer #1**

**This is a well-done paper that adds to the growing evidence on women’s mortality risk associated with parity and infecundity. I genuinely enjoyed reading this manuscript - the authors skillfully situate their study within the broader context of existing evidence, demonstrating a comprehensive understanding of the relevant literature. The analyses presented are methodologically sound and rigorous, and the findings offer valuable insights into understanding the complex dynamics of underlying reproductive health disparities by race/ethnicity. I commend the authors for their outstanding work and only have very minor suggestions that focus on clarifications to further strengthen the manuscript's coherence**

**Response:** We are grateful that Reviewer 1 thinks our study is well-done and enjoyed reading it. We very much appreciate Reviewer 1’s thoughtful comments and suggestions. We have considered all of them carefully as we have revised our manuscript.

**In the abstract and throughout the paper, the authors claim to examine the infecundity risk and parity by race – my suggestion would be to clarify that it is “by race/ethnicity” as they analyze the sample with non-Hispanic Black and White women (i.e., the combined race and ethnicity variable is used and therefore the term race/ethnicity is more accurate to signify the combination of both race and ethnicity into a single variable).**

**Response:** Thank you for this suggestion.

**Changes we made:** We replaced the term “race” with “race/ethnicity” or “race/ethnic” throughout the paper.

**The authors skillfully place their work in the context of previous evidence and present three aims addressed in their paper (p.11). I would like to see more specific hypotheses outlined as well (regarding the main associations and any moderation effects examined).**

**Response**: We appreciate Reviewer 1’s helpful comment**.**

**Changes we made:** In response, we made our hypotheses more explicit. Specifically, we added four hypotheses. We now list them at the end of the Specific Aims section of the paper (**Lines 258-278**), and refer to them in the Results and Discussion sections of the paper. The four hypotheses we added to the paper are listed below.

Regarding Fertility:

Hypothesis 1: We expect to find that Black women have a higher probability of infecundity, but, at the same time, an equal or higher mean number of births relative to White women.

Regarding Mortality:

Hypothesis 2: We expect to find a U-shaped parity-mortality association such that infecundity risk, low parity, and high parity, relative to 2 births, are positively associated with mortality.

Hypothesis 3: We expect to find that the life-course timing of childbearing is associated with mortality such that premarital birth, and younger and older age at first birth, elevate mortality risk.

Hypothesis 4: We expect to find that race-related differences in mortality risk are only partially explained by parity and its timing, such that Black women will have greater post-reproductive mortality risk than White women.

**Under “Adjustment Measures” (p.16) the authors explain that they assigned the never-married women the mean “Age at Marriage” instead of excluding them. What is the proportion of “never married” women in the sample? It is not clear why creating a categorical variable which would allow to more accurately capturing categories of age at marriage (e.g., never married, married before 20; etc.) was not chosen instead?**

**Response:** The number of never-married women in our sample is 212 (2.9%), as shown in Table 1. Their distribution is as follows:

0 Births	1 Birth	2 Births	3 Births	4 Births	5 Births	6+ Births	Total

Never Married

N	150	17	14	16	7	3	5	212

%	70.9	8.0	6.6	7.5	3.3	1.1	2.4	100

Originally, we considered various approaches for managing this small group of never-married women but hesitated to exclude them (see below). We decided to include them by assigning them the mean age at marriage and including a never-married flag to be included in the model. In response to this comment, we did two things. We re-ran models using the categorical approach suggested by the reviewer and we re-ran all of the models in Tables 2-5 on the sample that excluded the 212 never-married women. In both cases, the substantive results were the same as those we report in the paper. The results are robust. Because the polynomial functional form is roughly equivalent to the categorical form and is the most-utilized functional form in demographic studies of fertility, we retained our original approach in the revised paper. It is our preferred approach. We hope our due diligence in response to this comment is satisfactory to Review 1.

**Changes we made**: We now provide a citation to a study (**Line 449,** Guinnane et al. 2002) that uses a similar age at marriage polynomial approach. We also added at **Lines 384-392** the following:

Preliminary analysis of the joint distributions of *Age at Marriage* and *Children Ever Born* indicated that almost a third of never-married women had children and nearly 20% had two or more children. HRS/AHEAD women were not queried about non-marital unions, known to be associated with model variables of race/ethnicity, social class, health resources, and birth timing. Rather than exclude them, we assigned never-married women the mean *Age at Marriage* for the sample. flagging the substitution in the *Children Ever Born* count analyses. Sensitivity analyses that: (1) used age at marriage categories including never-married and (2) excluded all never-married women produced the same substantive results as those we report in the paper.

**In Table 1, please consider including the p-value of the difference between the two racial/ethnic groups.**

**Change we made:** We now include this.

**In Table 2, please include the 95% CI next to reporting odds ratios.**

**Change we made:** We now include this.

**The Discussion section is very well-written with the main findings outlined and discussed in sufficient detail. However, I would suggest enhancing the “implications for contemporary health research and policy” component indicated in the title of this paper. More specifically, based on the main findings of this study, what are the key policy implications in addressing racial/ethnic inequities in reproductive health in the US?**

**Change we made:** We address this in the final paragraphs, as follows, between **Lines 722 and 733**:

Continued research on the social and biophysiological determinants of infecundity is warranted. Additionally, the onset of health deterioration earlier in the life course more directly overlaps the reproductive years of Black than White women and reflects—among current as well as historical birth cohorts—lack of equitable access to societal resources, including health care.^67^ The results of this study indicate directions for ameliorative health policies, including the need to institutionalize universal, maternal care services that provide an added fourth trimester of post-delivery care. Past studies stressing maternal depletion, and current studies that locate most maternal mortality among today’s Black women in the post-partum period, all point to the need for post-delivery care to detect and remediate reproductive health problems.^67^ Additionally, greater translational application of current weathering/reproduction research is warranted. In addition to a need for universal, expanded reproductive care, there is need to restructure it, with less segregation/siloing of care outside of primary care in order to optimize maternal health prior to a first pregnancy and provide on-going monitoring of longer-run sequelae of pregnancy.^68^

**Reviewer #2:**

**This is an interesting paper which uses a two-step analytic process, first to separate voluntary from involuntary childlessness and therefore to estimate a risk of infecundity (involuntary childlessness) for individual women. The next step estimates the effect of this infecundity risk and of parity on later life mortality, and compares the results for black and white women in the US. The authors conclude that black women’s later life mortality risk is associated with their infecundity. The analyses seem appropriate (although I’m not an expert in these precise models and feel they could be better explained for the audience) and imaginative.**

We are pleased that Reviewer 2 found our paper interesting. We very much appreciate Reviewer 2’s thoughtful comments and suggestions. We have considered all of them carefully as we have revised our manuscript.

**Literature review and framing.**

1. **However, I think there are improvements the authors could make to the framing of the paper in order to make it more accessible. In particular, the focus on childlessness and post-reproductive mortality emerges rather slowly during the introduction and literature review. This could be made much clearer from the start and the reader would benefit from greater clarity in the literature review too.**

**Response:** We thank you for this observation and have revised the paper based on these comments (described below). However, please let us clarify that our investigation of the childlessness—post-reproductive mortality association is only one element in our study. We are as interested in the *overall relationship between parity and post-reproductive mortality.* Put differently, we are concerned that extant research on the parity—post-reproductive mortality relationship in the U.S. appears anomalous with respect to functional form (U-shape, linear, no association) relative to findings from a large body of non-U.S. studies because heterogeneity in childlessness status and race-related differences have not been adequately addressed. Because a growing literature from other Western nations consistently demonstrates a U-shaped parity—post-reproductive mortality relationship and U.S. studies show no consistent pattern, our first Introductory paragraph integrates U.S. studies into the larger field of parity—post-reproductive mortality research. We consider this contextualization essential to our project and think that the discussion in this first paragraph is an appropriate start to our paper.

**Changes we made.** This said, in response to Reviewer 2’s suggestion, we modified the Introduction in several ways to try to elevate the childlessness—post-reproductive mortality component of the parity—post-reproductive mortality relationship and clarify our literature review.

- In Paragraph 2, we added the phrase “parity—post-reproductive mortality” (**Line 65**) to reference its association with childlessness on **Line 66**.^18^ **Lines 67-69** now formally define infecundity and fertility (observed parity) based on definitions provided by (in an added citation) McFalls (2009). Descriptions of the childlessness-mortality association continue in **Lines 70-71** and **80-83.**
- Paragraph 3, **Lines 86-91**, address childlessness and post-reproductive mortality, although the focus shifts to estimation of infecundity.
- In the next section of the paper starting at **Line 102 and Lines 104-108**, we more fully describe the importance of childlessness in shaping the parity—post-reproductive mortality functional form. Specifically, we emphasize Hurt et al.’s (2007) finding that “in the thirteen historical cohorts studied, mortality was highest among childless and low-parity women and declined with parity; the contemporary birth cohorts more often exhibited U- or J shaped parity-mortality curves.”
- The remainder of the Childbearing Context section (**to Line 165**) develops why deeper investigation of childlessness, at least in the U.S. case, is important.

2. **I also feel that the paper would benefit from closer attention to the historical context that the study cohorts were going through. In particular, I would have liked some recognition that the cohorts of women studied (born 1920-1941) were the cohorts going through childbearing during the baby boom. We know that such women had unusual marriage and childbearing patterns compared to those before and after. How might this affect conclusions?**

**Response:** We appreciate these comments as they astutely highlight the importance of time (“period”) as context, and the importance of changing marriage and childbearing patterns across time.

**Change we made:** In response to these comments, we have added the following text on **Lines 115-123**:

…Women in these historical birth cohorts shared atypical marriage and childbearing patterns; many could have given birth between 1946-1964, thereby contributing to the historical Baby Boom. Unlike women in contemporary birth cohorts, they more likely married, married prior to age 25, preferred to bear at least one child, had higher completed fertility, and primarily gave birth within marriage.^7,29^ Indeed, one advantage of studying historical populations marked by a high prevalence of early marriage and marital childbearing, is that the parity—post-reproductive mortality relationship is less likely to be masked. Another advantage is that the potential selectivity surrounding marital childbearing that occurs in contemporary birth cohorts is minimized.^7,12^

This addition also answers the question: **How might this affect conclusions?** Our sample, comprised of women with a high prevalence of marriage, early marriage timing, and marital childbearing should facilitate assessment of the parity-mortality relationship, at least from a bio-evolutionary perspective.

3. **Were there differences in the experience of the baby boom that affected black and white women differently? It might be easier to conceptualize these baby boom generations as an atypical episode rather than in between two ‘cross-overs’ (lines 123-126).**

**Response:** We agree that marriage and childbearing patterns during the Baby Boom were historically atypical, with marked differences in patterns among Black and White women. Black women, until the mid-twentieth century, married at earlier ages, had higher marriage/remarriage rates than White women (were more exposed to marital fertility), and, at least 1900-1910, less likely practiced marital fertility control. Reynolds Farley and others found the high childlessness rates in 1880-1940 U.S. birth cohorts of Black women, born primarily in the Jim Crow South, worthy of note because their marital and childbearing patterns supported high fertility. As we noted above, in response to Reviewer 2’s second comment, we now acknowledge in the text just prior to the sentences referenced in this comment that all women in our study passed through some period of their childbearing career during that Baby Boom era, and that marriage and childbearing patterns during this historical period were atypical.

**Changes we made:** To address Reviewer 2’s additional observations and suggestions, we have also rewritten **Lines 129-136** as follows:

Fertility control practices, however, diverged more by race/ethnicity than region.^21,22^ Southern White women by 1900, like White women in all U.S. regions, show evidence of fertility control by both limiting marriage (nuptiality) and marital fertility.^21,29^ Southern Black women, compared to all White women, more likely married, remarried, had higher fertility rates, and less-likely limited marital childbearing.^20-22,31^ Their reproductive practices reflected their distinctive childbearing contexts: about 95% of U.S. Black women in 1900 (dropping to 75% in 1940) lived in the South and larger family sizes benefited their predominantly agricultural, but not land-owning, household economies.^32,33^

**Unchanged:** The intent of original Lines 123-126 was to provide statistical evidence about U.S. Census-based race/ethnic differences in childlessness rates in 1880-1940 birth cohorts. [The topic sentence of the paragraph that includes original Lines 123-126, (now Lines 140-144) is that *Black women born between 1880 and 1940 had remarkably higher rates of childlessness than White age peers (birth cohorts 1880-1940)*.] The published demographic analyses behind the birth cohort crossover statements are sound. Our paper examines race differences in childlessness in the HRS birth cohorts that overlap them. Thus, we consider this essential contextual information and have retained it in the paper. Both period effects of Baby Boom-related fertility patterns and birth-cohort effects of rising and falling childlessness can be and are true.

4. **It would also be good to recognize (eg line 71) that historically the most important driver of childlessness was celibacy (non-marriage). Fecund women were likely to have been selected into marriage through pre-marital pregnancy.**

**Response:** Thank you for this astute comment. We added this substance of this comment to **Line 73**.

**5. Sometimes an awful lot of work is done by the term ‘infecundity’ (eg Line 130: the use of ‘infecundity’ here seems to represent a composite of everything excluding voluntary control, but this includes a wide range of factors).**

**Changes we made: we now define infecundity, early in paper (Lines 67-70);** in Line 130 (now **Line 148**), we changed “infecundity” to “poor reproductive health.” Much of the rest of the paper addresses infecundity probability or risk based on the statistical estimation of infecundity.

6. **How can you tell the differences between infecundity (as in the biological incapacity to conceive), exposure (eg through marriage/partnership), and voluntary childlessness (use of contraception)?**

**Response:** We understand this reference to Bongaarts proximate determinants of fertility: marital exposure, voluntary control, and biophysiological factors. As noted above, the historical cohorts we study, compared to contemporary ones, had high marriage rates (almost universal in HRS data, even without accounting for/reporting the alternative of cohabitation). The more difficult distinction involves women’s likelihood of involuntary and voluntary childlessness, with implications for post-reproductive mortality. [Continued below]

**Also: (We have moved this comment up)**

**Analytic strategy.
7. I would have appreciated more explanation of how the combination of the logistic and count processes can distinguish voluntary and involuntary childlessness. Does this depend on an assumption that you can predict the proportion of women who are voluntarily childless from the subsequent parity distribution. Why would this be so? How do these models differ from ‘cure’ models?**

**Response**:  These models are well established in demographic fertility research. We cite two methodological papers (Poston and McKibben 2003; Miranda and Trivedi 2020) and two application papers (Guinnane et al. 2002, 2006). The method can empirically distinguish groups of women in our data—if present—with a high probability of childlessness due to involuntary processes (an infecund group), and with a low probability of being in an infecund group. The latter group of women might also have zero children but they more statistically resemble the profile for fecund women. Of course, we cannot adjudicate between the involuntary processes in HRS data which, according to Bongaarts, might include sterility, miscarriage, intrauterine death, and infant mortality.

Zero inflation models are not like cure models which, from our understanding, longitudinally follow populations over time, thereby distinguishing those who do and do not experience an event, such as recurrence of disease or having a child. The HRS sampled respondents past childbearing age and so we essentially estimate completed fertility in a cross-section. The zero-inflation method is useful if one suspects that the “0” responses in the data derive from different groups. For example, a count model of *number of cigarettes smoked last week/in a usual week* or *number of drinks last week/in a usual week* in population-based sample would have lots of zeros but the zero category would be inflated due to combining different populations/groups: some zeros would be reported by smokers/drinkers who did not smoke/drink in the period of interest, while other zeros would be reported by non-smokers and non-drinkers. It is useful to distinguish the groups.

**Changes we made:** We changed the Analytic Strategy description **Lines 418-456** as follows:

The first dependent variable in our study, *Number of Children Born*, is a count. Count regression methods are recommended for modeling count dependent variables as they have non-normal distributions. A first-choice model, a Poisson count model, assumes relative homogeneity in a study population (assumes that count equation variance is equal to its mean), although study populations’ characteristics might vary. Statistical tests of Poisson model fit to the data allow this assumption to be rejected. If so, (e.g., if there is a great deal of error variance or dispersion in the Poisson model), a next step is to estimate a negative binomial count model that relaxes the Poisson variance-equal-to-the-mean assumption.

Preliminary analysis of our sample showed that 10.5% of Black women and 8.6% of White women had zero births. We knew that, in theory, childlessness (zero births) among sample women might have reflected prior biophysiological (infecundity) or voluntary limitation processes. Moreover, if two different underlying processes (infecundity vs. childbearing control) had produced zero births, two different groups of childless women would be present in our sample. The childless women from different groups could “inflate” the zeros in the fertility count. A negative binomial count model, used to relax the assumption of population homogeneity, might still not provide an adequate or best fit to the data.

That different underlying processes can produce different groups of childless women poses a problem of distinguishing the infecund from voluntarily childless nulliparous women. Fertility studies address this problem by additionally testing fit to the data of zero-inflation Poisson (ZIP) and zero-inflation negative binomial (ZINB) count models (Guinnane et al. 2006; Poston and McKibben 2003). These models estimate the sample women’s probabilistic membership in one of two possible latent groups: an “always-zero” or infecund group and a group where women might have zero births but do not statistically fall into the always-zero profile. ^16,19,22,61^ Specifically, zero inflation models estimate a count of women’s observed number of children born as a function of two distinct processes: (1) a logistic (or probit) process that distinguishes a possible latent, always-zero group of probabilistically infecund women; and (2) a count process that estimates the parity distribution among probably fecund women, also allowing for zero births. Zero-inflation models allow evaluation of the hypothesis that there is a latent always-zero (i.e., infecund) class of women in the data.

Using Proc Genmod, in SAS 9.4, we modeled women’s *Number of Children Born* as a function of: age; age at marriage (polynomial), marital duration (Guinnane et al. 2002), and number of siblings and race/ethnicity. We initially used Poisson count models. Finding considerable dispersion, we additionally examined negative binomial and ZIP and ZINB models. We compared, across regression models, model fit to the data using: ratios of Pearson chi-square and deviance scores to degrees of freedom to test overdispersion; and Clarke tests to determine the presence/absence of a distinct latent always-zero class of probabilistically infecund women. We also examined overall model fit and parsimony of fit by using plots of predicted count distributions against the observed count and BIC scores, respectively.

8. **Demographers have specific meaning for fecundity and fertility, but these terms are not used in the same way by every field or by the general public. Clarity in how the authors are using them would be helpful.**

**Response:** We appreciate the importance of this.

**Changes we made:** We now define both (in)fecundity and (in)fertility on **Lines 67-70**. Elsewhere in the paper, **(Lines 77-80**), we indicate a definition of infecundity, as related to women’s biophysiological likelihood of bearing children. But most of the paper either references the probability of infecundity (involuntary childlessness) or refer to infecundity as a statistical relationship, based on empirical models: “the probability of infecundity,” “infecundity risk” or as the variable *Infecundity.*

9. **This is also relevant for lines 169-171: Because most measures of fertility include only live births, the ‘healthy pregnant woman effect’ – surely this selection is not just about healthy pregnant women, but healthy women who produce live births. Similarly on line 191, ‘the physiological resilience to the fecund’ should be ‘the physiological resilience to be fertile’.**

[**Note: Original Lines 168 to 173 state**: 168 …. Together, these factors may account for a

169 lack of an expected trade-off pattern. As such, a default selective “healthy pregnant woman

170 effect” may underlie observed historical population patterns: only reproductive-aged women

171 with the physiological resilience to be fecund *and* survive exposure to infectious diseases,

172 malnutrition, and other health risks, including past pregnancies, reach higher parities and post

173 reproductive ages.^12,25^]

**Response: This material is now between Lines 187-190.** We agree with and very much like this distinction, that the “healthy pregnant women effect” is more about women who produce live births. However, we were careful in Original Lines 169-197 to only reference the term in passing, using quotes. The rest of original Lines 170 to 173 restated this logic in the manner here suggested: “only reproductive-aged women with the physiological resilience to be fecund *and* survive exposure to infectious diseases, malnutrition, and other health risks, including past pregnancies, reach higher parities and post-reproductive ages.”

**Change we made:** Using a definition of fecundity as reproductive ability and fertility as actual childbearing (McFalls 2009) we now substitute (**Lines 187-190**):

“only reproductive-aged women with the physiological resilience to have reproductive ability *and* survive exposure to infectious diseases, malnutrition, and other health risks, including actual childbearing, reach higher parities and post-reproductive ages.”

**Unchanged:** We agree with the pregnancy-live birth distinction but did not coin the “healthy women pregnancy effect” term and feel the need to continue to cite it, in quotes, simply to give credit to those who proposed the innovative logic that it represents.

10. I**’m surprised that the proximate determinants of fertility framework is not mentioned in the text (Bongaarts is referenced in the literature) - this might help with the conceptualisation.**

**Response:** We are, of course, well aware of Bongaarts’ proximate determinants framework and it has informed our thinking considerably. We cite Bongaarts in several places. However, our data and modeling can only differentiate probabilistically involuntary and voluntary childlessness. This full framework and that used by McFalls introduces factors that we cannot test, such as contraceptive use. We are, however, using such frameworks in a parallel work-in-progress that uses a database whose variables include: number of pregnancies, number of live births, and contraceptive use. We hope interested readers follow our citations of Bongaarts and the *Annual Review of Sociology* pieces by McFalls and Sweeny and Raley covering these frameworks.

11. **In ‘Biophysiological factors’ I’m surprised that the protective effect of breastfeeding on the risk of breast cancer was not mentioned.**

**Response:** This study examines all-cause mortality, modeled as a function of parity, its life-course timing, age, and race/ethnicity, with adjustments. We very much agree that it is important to examine disease-specific pathways between parity and mortality. For example, Henretta’s (2007) study using the HRS 1931-1941 birth cohorts examines the parity—post-reproductive mortality relationship and also health conditions, finding breast cancer an important pathway. More-recent studies report cardiovascular and diabetic pathways between parity and mortality. We decided to limit discussion in this paper to all-cause mortality. We would not only need to describe cancer risk, but the importance of other pathways; we would need to add several paragraphs to our literature review to cover this material even though we do not examine disease-specific mortality outcomes. We are, however, currently examining disease-specific pathways linking parity to later-life mortality, including cancer, in follow-up work using the HRS.

**Line 111: ‘natural fertility’ should be defined for those who are not familiar with the term**

**Changes we made:** We modified the term in **Lines 127-128** (original Line 111) with “populations that do not voluntarily control their fertility” and cited Bongaarts as well as Tolnay.

12. **In the ‘Socio-economic factors’ section (lines 175-228), some of the factors mentioned explain the increased risks associated with low parities, and some explain the increased risk of high parities: it would be helpful to the reader if these were distinguished.**

**Response:** The Section lists the following socio-economic factors: maternal depletion, weathering, parental health behaviors, social integration/child support, and national old-age support. We are not aware of studies linking maternal depletion, weathering to specific numbers of births (low parity vs. parity).

We note, **Lines 229-230** that: health behaviors of those with higher-fertility become more 228 health-protective with increased childrearing experience.

We are aware of studies linking receipt of national old-age support to reduced parity but most of our sample, as noted above, contributed to the Baby Boom. In the case of social integration, the association between number of children born and own-child support (the way post-reproductive longevity is theoretically produced) among early- to mid-twentieth century U.S. elders is debated by demographers. For example, much multigenerational coresidence in pre-1940s Census data reflected the midlife childbearing ages of women more than child caregiving.

**Change we made:** This said, we added (**Lines 238-240**):

Some of the factors noted above (e.g., childhood poverty, poor living standards) are likely

associated with both childlessness and high fertility, albeit though different mechanisms.

They also are likely to vary in relation to race/ethnicity.

13. **Line 260: ‘The RAND documents question changes and …’ I had to read this several times before I saw documents as the verb ad question as a noun rather than the other way around. I suggest a rephrase.**

**Change we made:** We see the problem and changed this (**Line 293**) to: “The RAND provides harmonized responses…”

14. **Data. Regarding the observed parity and timing indicators (lines 301-311), age at first birth is calculated using the age of participant and the age of their first child. This implies that respondents were asked for the ages of all their children, including the age that any who had died would be had they lived. This might be subject to more error than the ages of living children, and it would be good to have an assessment of the effect of this on results.**

**Response:** Although the HRS did query respondents about their number of children, it did not ask them about the age(s) of all children born or list dates of all children born. Rather, it only asked them to report the age of their oldest and youngest **living child with whom they were in contact** in the baseline interview. The RAND family database harmonized this information. The codebook notes:

Age of the respondent’s youngest kid and oldest kid…variables are derived from the best guess kid’s age in the Respondent-kid file. We noticed that some of the ages are over 80 years old.

Page 247, RAND HRS Family Data 2018 (V2). Santa Monica, CA (July 2023).

<https://hrsdata.isr.umich.edu/sites/default/files/documentation/codebooks/1690235656/randhrsfam1992_2018v2.pdf>

**Change we made:** To clarify this we have added, **Lines 337-339:**

(The RAND family database provides information only about each participant’s oldest and youngest currently-living child with whom they were in contact at the intake interview, not all children born).

15. **Treatment of missing data: did you do sensitivity analyses to examine how much the use of mother’s education when father’s was missing?**

**Response:** Yes, we created a flag for the fill-in cases (196 cases). This was not significant in any preliminary model testing.

16. **Lines 401-403: explain the significance of this correlation.**

[Original Lines 399-403: To preview findings, the zero-inflation negative binomial (ZINB) functional form best fits the data; it identifies women in our sample with a statistically high probability of selection into an always-zero parity class. In the sample, the correlation between the probability of selection into an always-zero parity and observed zero parity (childless) is .27; the R2 (% of variance explained) is 7.3% (0.27 x 0.27 = 0.073).]

**Response:** The lines (now **Lines 458-461**) report the correlation of the *Infecundity probability,* obtained from the always-zero portion of ZINB model, with a dichotomous variable, zero births as reported in the HRS. We provided this information because the mortality analyses examine observed zero parity alone (Models 1), and observed parity with *Infecundity* (Models 2). The correlation suggests that the measures are not collinear.

**Results.**

**17. Fertility models: Age and cohort. Age (eg on line 448) presumably means age at survey, but which round of the survey? It might be less confusing to present this as cohort. If it is not cohort, what is its relevance? On line 470-1 you say that ‘age and marital age represent biological parameters of reproductive physiological maturation’ – I’m not sure how age at survey would do that, or does this now refer to age at birth?**

**Response: Line 349** reports that *Age* indicates participant age at the first interview, taken from the HRS Tracker file. The HRS Tracker file contains the best possible information about age; the tracker-file age at baseline measure follows participants until they leave the study and is often used in event history studies where age is the time axis.

**18. Results are conditional on surviving to the survey date: how might differential survival up to that point affect results?**

**Response:** We address this in great detail in the Discussion Section, **Lines 685-706**.

**19. Descriptive information (Table 1) indicates that the parents of blacks were better educated than the parents of whites, but the black respondents themselves were worse educated. This seems unexpected – is there some sort of strange selection operating?**

**Response:** We appreciate your capturing this; the percentages were correct but we inadvertently mislabeled (flipped) the rows reporting parent schooling variables for all and for White and Black respondents. **We have corrected this in Table 1.**

**Parent:	All women	White women	Black women	Black women: own schooling**

Less than 8^th^ grade	24.2	21.5	37.1%	Less than HS	44.6%

8^th^ grade or more	68.0	72.1	49.3%	High school	42.9%

Greater than HS	12.6%

Regarding comparing Black women’s parent to their own schooling, we do not believe such a comparison is possible, due to different endpoints. About 44% of HRS Black women did not complete high school, which is more than the 37% of their mothers with less than 8^th^ grade education. However, mothers with 8^th^ grade or more may have stopped at 9^th^ or 10^th^ grade and still have less education, on average, than the HRS Black women who dropped out of high school. One cannot know this from HRS data.

**20. Count model: presumably this refers just to women who are not infecund/infertile? This should be made clearer.**

**Response:** In the Analytic Strategy section, we note that the count portion of the model is based on the distribution of number of births, after accounting for women’s probability of belonging/not belonging to a latent always-zero class. It is the probably fecund group of women.

**21. More guidance is needed on how to interpret the models, for example the beta coefficient in Table 2. Why not also give the exponentiated values in the count bit of the Table (particularly as these are reported in the text). In the ‘always-zero’ bit of the Table, could the odds ratio be shown?**

**Changes we made:** We have added both IRRs (exponentiated values) to count coefficients and odds ratios/95% confidence intervals to the always-zero portion of Table 2.
